# *Cyclospora* Genotypic Variations and Associated Epidemiologic Characteristics, United States, 2018–2021

**DOI:** 10.3201/eid3102.240399

**Published:** 2025-02

**Authors:** John Shen, Vitaliano A. Cama, David Jacobson, Joel Barratt, Anne Straily

**Affiliations:** Oak Ridge Institute for Science and Education, Oak Ridge, Tennessee, USA (J. Shen); Centers for Disease Control and Prevention, Atlanta, Georgia, USA. (J. Shen, V.A. Cama, D. Jacobson, J. Barratt, A. Straily)

**Keywords:** *Keywords:* Cyclospora, parasites, food safety, outbreaks, clinical manifestations, cyclosporiasis, genotyping, molecular epidemiology, Texas, Florida, New York, United States

## Abstract

Seasonal cyclosporiasis outbreaks occur in the United States every year. To better understand the disease, the Centers for Disease Control and Prevention developed a novel genotyping system that successfully clusters nonclonal eukaryotes. We examined temporal-geographic distributions of *Cyclospora* cluster consensus genotypes (CCGs) and applied regression analyses to identify correlations between *Cyclospora* spp. parasites and clinical manifestations or epidemiologic risk factors, using data collected during 2018–2021. No CCG was uniquely associated with or consistently detected in a state during the study, suggesting that cyclosporiasis in the United States is likely caused by frequent parasite introductions. We identified positive associations between infection with *C. ashfordi* and *C. cayetanensis* and consumption of specific produce items: cilantro, mango, and onion for *C. ashfordi* and iceberg lettuce, carrot, and cauliflower for *C. cayetanensis*. Our findings can guide future research into public health interventions aimed at reducing the burden of cyclosporiasis in the United States.

*Cyclospora* spp. are foodborne parasites that cause seasonal outbreaks of cyclosporiasis in the United States (*1*). Cases of this diarrheal disease are often sporadic and geographically dispersed; those characteristics, combined with the lengthy lag period between illness onset and patient interview (typically 4–6 weeks), make it difficult to identify the food vehicles of infection (*2*). Although cyclosporiasis has been reported year-round in the United States, most cases occur during May–August (*3*). Molecular typing, in conjunction with epidemiologic methods, has increased our understanding of disease transmission dynamics for other pathogens (*4*). For cyclosporiasis, the Centers for Disease Control and Prevention (CDC) developed a *Cyclospora* genotyping system, Cybernetic Clustering Of Nonclonal Eukaryotes (CYCLONE) bioinformatic workflow (*5**–**7*), that uses Illumina (https://www.illumina.com) sequence data generated from a set of 8 PCR-amplified *Cyclospora* genetic markers as input and computes a pairwise distance similarity matrix that is hierarchically clustered to yield clusters of genetically similar samples (*8*).

Application of CYCLONE previously revealed 2 distinct species of *Cyclospora*, *C. cayetanensis,* and *C. ashfordi*, as agents of cyclosporiasis in the United States (*7*). Those species are distinguished at the 360i2 nuclear locus and several other loci throughout the *Cyclospora* genome not included in CYCLONE; alleles are exclusive to each species (*7*). That study reported geographic and temporal trends during 2018–2020, when *C. cayetanensis* accounted for an estimated two thirds of documented cyclosporiasis in the United States and was generally a more common cause of illness in northern and midwestern states, whereas Texas and Florida had greater proportions of *C. ashfordi* infections (*7*).

The previous observations required deeper analysis, which is the focus of our study. To more precisely track genetic types over different time periods and determine the possibility for locally established foci of infection, we developed a novel approach for cluster categorization, termed cluster-consensus genotypes (CCGs). First, we analyzed observed temporal and geographic patterns by CCG among specimens submitted for *Cyclospora* genotyping during 2018–2021 to better understand annual variation of *Cyclospora* CCGs by year and reporting state. Second, we analyzed clinical, biologic, or epidemiologic features that could be associated with the different species of *Cyclospora*, as has been observed for species of *Cryptosporidium* (*9*). We anticipate that our results will serve to bolster our understanding of cyclosporiasis in the United States.

The human subjects coordinator at CDC’s National Center for Emerging and Zoonotic Infectious Diseases reviewed this project and deemed it a nonresearch public health surveillance activity. We conducted the activity consistent with applicable federal law and CDC policy (45 C.F.R. part 46; 21 C.F.R. part 56). 

## Methods

The study is based on 2,770 *Cyclospora*-positive fecal samples successfully genotyped by CDC during January 2018–December 2021 as available via National Center for Biotechnology Information under BioProject no. PRJNA578931 (*5*,*10*,*11*). We excluded from the analyses 1 fecal sample that was positive for *C. henanensis* and sequenced as part of the genotyping reference set (*7*) and samples for which the state of origin could not be ascertained.

### Determination of *Cyclospora* CCGs

**Figure 1:**
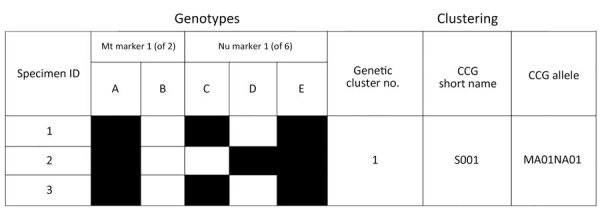
Schematic representation of a cluster consensus genotype *in study of* *Cyclospora* *genotypic variations and associated epidemiologic characteristics* United States, 2018–2021. Genotypes are derived from 8 markers, 2 Mt and 6 Nu; this schematic representation is based on 1 Mt and 1 Nu marker, where the haplotypes for this Mt marker are A or B, and the Nu haplotypes are C, D, or E. The Centers for Disease Control and Prevention *Cyclospora* genotyping system, Cybernetic Clustering Of Nonclonal Eukaryotes (CYCLONE) bioinformatic workflow, was used to determine the genetic similarity and clustered specimens 1, 2, and 3 in genetic cluster 1. Specimens 1 and 3 have genotype ACE, and specimen 2 has genotype ADE. Because genotype ACE is present in ≥50% of samples, it is the CCG for cluster 1, and its short name for this example is S001. The corresponding allele for this specific CCG is MA01 (Mt marker A) NA01 (Nu markers C and E). CCG, cluster consensus genotype; ID, identification; Mt, mitochondrial; Nu, nuclear.

We submitted sequencing reads to CYCLONE for genotyping and used specimens with haplotypes detected for >5 of the 8 markers to generate genotypes and a pairwise distance matrix in CYCLONE. We clustered the resulting matrix hierarchically to produce a tree, which we dissected into discrete clusters of closely related specimens (*8*,*12*). Within each cluster, the genotype found in >50% of specimens became the representative CCG. Because CCGs are an emergent property of the clustering process, the individual genotypes within a cluster might vary slightly from the representative consensus genotype despite sharing most of their core haplotypes. For this study, we identified each CCG with the letter S and a 3-digit number (e.g., S001) (Figure 1). We analyzed proportional distribution of the 2 *Cyclospora* species on the basis of the submitting state and year of submission. A similar analysis at the CCG level looked at the 5 most frequently detected CCGs within states across the 4 years of the study and presented results as heatmaps. Within our study dataset, some genotyped specimens were previously epidemiologically linked to defined outbreak clusters, but others were not. Therefore, we conducted the CCG analysis first on all available samples (linked and not linked to an outbreak), and then once again only with specimens not linked to epidemiologically identified outbreak clusters, to determine if similar patterns were observed among non–outbreak-related samples.

### Associations between Species and Disease Symptoms or Reported Produce Consumption

We filtered the dataset to keep only genotypes with associated epidemiologic data and complete genotype data at the 360i2 locus that was previously described as a species-defining allele for human-infecting *Cyclospora* (*7*). When a patient had >1 specimen genotyped, we kept the sample with the most complete genotype. However, if a patient’s samples had discordant genotypes (e.g., for patients who became infected with different species on separate occasions), we excluded all patient samples from the analyses.

We used epidemiologic data from Cyclosporiasis National Hypothesis Generating Questionnaires (CNHGQs), completed by US cyclosporiasis patients during routine public health surveillance, to determine statistical associations between each species of *Cyclospora* and patient-reported clinical manifestations and produce consumption. The CNHGQ includes questions on patient demographics, state of residence, travel, clinical manifestations, and food consumption in the 2 weeks before symptom onset. We dichotomized responses about symptoms or consumption of food, using yes for yes/maybe responses and no for don’t know/no responses. We used a patient’s home state of residence as a proxy for the site of infection acquisition. Because domestic or international travel affected the validity of this proxy variable, we included travel variables in a regression model described in the next section. The CNHGQ includes food parent variables (e.g., bell pepper); if those elicit an affirmative response, the next question is for specific subset exposures within that parent variable (e.g., red bell pepper, orange bell pepper). We selected food parent variables for analysis and not the subset questions, which frequently had missing responses. *Cyclospora* outbreak investigations identified whether a person belonged to a specific epidemiologic cluster, in which >2 patients were linked to the same source of infection (i.e., food vehicle, store, or restaurant); such clustering was also factored into the final regression model.

### Statistical Analysis

We described continuous variables by mean and SD or median and interquartile range and categorical variables by count and percentage. We assessed potential associations between *Cyclospora* species and epidemiologic features using Welch 2- sample *t*-tests for continuous variables and Pearson χ^2^ or Fisher exact tests for categorical variables. If we could not assume a normal distribution for a continuous variable, we performed a Kruskal-Wallis rank-sum test instead. We presented missing data as the count of patients without a recorded value for a characteristic. We determined p<0.05 as statistically significant.

We used a generalized estimating equation (GEE) with a homogeneous exchangeable/compound symmetric covariance and correlation matrix structure to estimate associations between statistically significant variables from univariate analyses and *Cyclospora* species. This method enables clustering of outcomes as a result of genetically similar pathogens being connected with a common food vehicle in routine epidemiologic surveillance. Because a previous study (*7*) observed statistically significant temporal and geographic differences between *Cyclospora* species, we included both those characteristics in the model. The temporal component comprised 2 variables: year of detection and day of the year (i.e., ordinal date) of symptom onset. Geographic variables included indicators for whether patients lived in Texas or Florida (2 states with higher proportions of *C. ashfordi* infection in the previous study [*7*]), whether they traveled out of state, and whether they traveled out of country. We added food and demographic variables to assess if statistically significant differences from bivariate analyses would be maintained after adjusting for covariates. We excluded patients missing any data for selected predictors. We performed all data cleaning, variable transformations, and statistical analyses using R statistical computing and graphics software (The R Project for Statistical Computing, https://www.R-project.org). We used the geepack package (*13*–*15*) to fit the GEE and ggplot2 (*16*) to produce the plots.

## Results

### Temporal-Geographic Distribution of *Cyclospora* Species and CCGs

**Figure 2:**
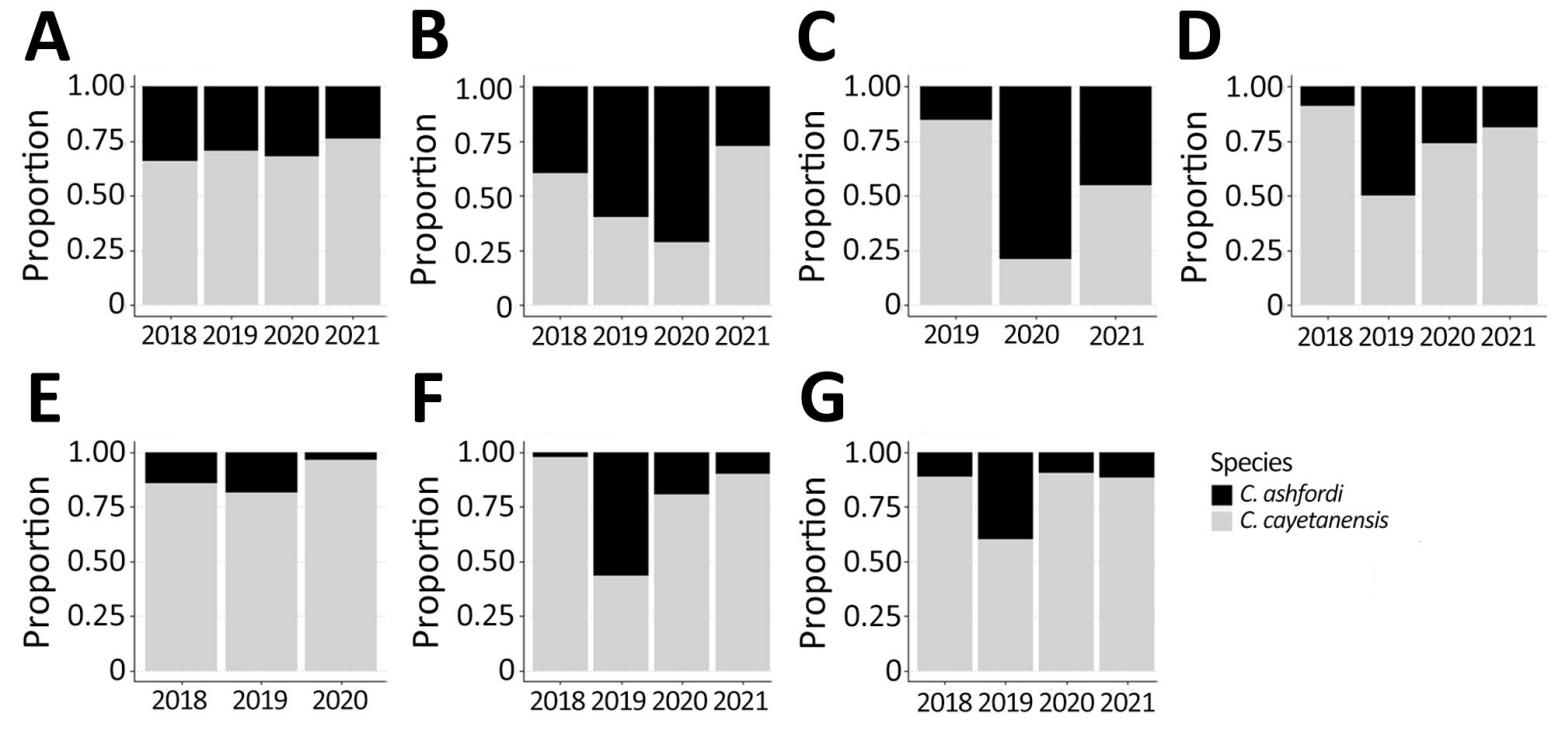
Proportion of *Cyclospora cayetanensis* and *C. ashfordi* in 7 states with highest number of specimens *in study of* *Cyclospora* *genotypic variations and associated epidemiologic characteristics* United States, 2018–2021. A) New York; B) Texas; C) Florida; D) Illinois; E) Iowa; F) Wisconsin; G) Minnesota. No specimens were submitted for genotyping from Florida in 2018 or from Iowa in 2021.

Of the 2,770 specimens processed through CYCLONE, we excluded 9 *Cyclospora* specimens (0.3%): 1 reference isolate from China and 8 specimens for which the state of origin could not be ascertained. The evaluable 2,761 specimens originated from 33 states. The 7 states with the most isolates genotyped over the 4-year study period were New York (n = 651), Texas (n = 551), Florida (n = 222), Illinois (n = 212), Wisconsin (n = 224), Minnesota (n = 204), and Iowa (n = 185) (Figure 2; Appendix).

Overall, 67.1% of samples were *C. cayetanensis* and 32.9% were *C. ashfordi*; those percentages varied by state and year (Figure 2). In New York, *C. cayetanensis* accounted for 65.7%–76.3% of all genotyped specimens. In Iowa, the prevalence of *C. cayetanensis* fluctuated from 81.5% in 2018 to 96.6% in 2020; no specimens were genotyped in 2021. In Texas, we identified *C. ashfordi* in 71.4% of specimens submitted in 2020, dropping to 27.3% in 2021. In Florida, the prevalence of *C. ashfordi* ranged from 15.4% in 2019 to 78.9% in 2020. *C. cayetanensis* was more prevalent in Illinois, Wisconsin, and Minnesota except for 2019, when the prevalence of *C. ashfordi* increased to 50.0% in Illinois and 56.7% in Wisconsin.

**Figure 3:**
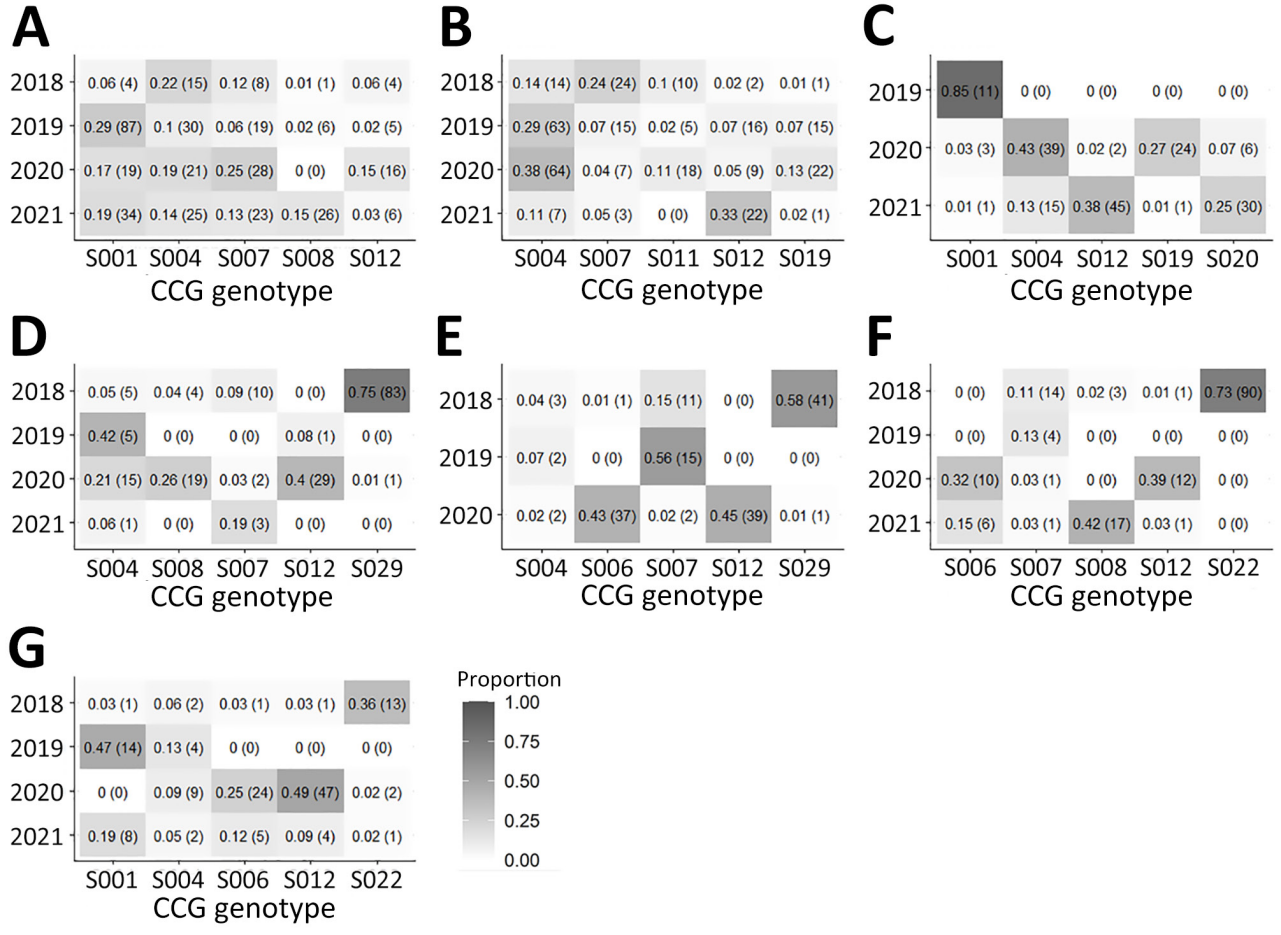
Heatmaps illustrating the proportions of the top 5 most prevalent CCGs in each of the 7 states with highest number of specimens *in study of* *Cyclospora* *genotypic variations and associated epidemiologic characteristics* United States, 2018–2021. A) New York; B) Texas; C) Florida; D) Illinois; E) Iowa; F) Wisconsin; G) Minnesota. Values within each box represent the percentage of the total number of specimens within a state for a given year. Numbers in parentheses represent the corresponding number of specimens. Darker shades represent higher proportions. The total number of specimens submitted per year is as follows: New York, 67 (2018), 297 (2019), 110 (2020), 177 (2021); Texas, 98 (2018), 219 (2019), 168 (2020), 66 (2021); Florida 0 (2018), 13 (2019), 90 (2020), 118 (2021); Illinois, 111 (2018), 12 (2019), 73 (2020), 16 (2021); Iowa, 71 (2018), 27 (2019), 87 (2020), 0 (2021); Wisconsin, 123 (2018), 30 (2019), 31 (2020), 40 (2021); Minnesota, 36 (2018), 30 (2019), 95 (2020), 43 (2021). CCG, cluster consensus genotype.

In this study we identified 33 CCGs; 20 belonged to *C. cayetanensis* and 13 belonged to *C. ashfordi* (Table 1; Appendix Table 2). The annual distribution of CCGs (Table 1) varied considerably year-to-year. The predominant CCG in 2018 was S029 (27.6%, *C. cayetanensis*), in 2019 was S004 (17.5%, *C. ashfordi*), in 2020 was S012 (23.0%, *C. cayetanensis*), and in 2021 was S012 (19.7%). Of note, CCG S012 represented only 1.4% of specimens in 2018, but its proportion increased in 2020 to 23.0%. Furthermore, we noted high heterogeneity in CCG prevalence within each state over the study period. In some instances, CCGs that were abundant one year were nearly absent in subsequent years (e.g., S029 in Illinois) (Figure 3). Such shifts were less extreme in states like New York, Texas, and Florida that had more cyclosporiasis cases. In general, no specific CCG was uniquely associated with a state, and most CCGs were not detected in similar proportions over the study period. Similarly, no CCG was uniquely associated with a state or year in the analysis of the 2,044 specimens (74.0%) not related to outbreak clusters (Appendix Figure).

**Table 1:** Classification of CCGs by *Cyclospora* species and distribution by year *in study of* *Cyclospora* *genotypic variations and associated epidemiologic characteristics*, United States, 2018–2021*

Species	CCG ID	2018	2019	2020	2021
*C. cayetanensis*	S001	2.3	17.3†	3.1	10.4
	S002	1.6	2.7	0.3	2.6
	S003	2.0	2.7	0.6	3.4
	S005	1.8	3.5	0.5	2.4
	S006	1.6	0.4	16.9†	4.4
	S007	14.2	7.1	8.3	9.0
	S008	1.3	1.5	0.0	11.4
	S009	2.5	1.8	0.5	1.1
	S012	1.4	3.1	23.0†	19.7
	S013	0.4	0.9	0.3	1.3
	S014	0.4	1.5	0.0	0.8
	S015	0.7	1.1	1.0	0.7
	S018	1.1	1.3	0.1	1.3
	S021	0.7	3.9	0.6	1.5
	S022	20.2†	0.5	0.5	0.8
	S025	0.5	1.2	1.3	1.0
	S027	0.4	1.1	1.0	1.5
	S028	1.1	1.7	0.5	1.5
	S029	27.6†	1.3	0.4	0.0
	S033	0.0	0.2	4.0	0.3
*C. ashfordi*	S004	7.2	17.5†	21.4†	9.6
	S010	1.8	0.7	0.1	0.7
	S011	2.2	1.5	2.6	0.5
	S016	0.5	5.9	0.0	0.2
	S017	0.5	1.6	0.5	0.8
	S019	0.5	3.5	6.8	0.5
	S020	3.2	2.1	2.1	7.7
	S023	0.5	8.3	0.5	0.7
	S024	0.4	1.6	0.4	2.1
	S026	0.4	0.4	0.0	0.7
	S030	0.4	0.7	0.6	0.5
	S031	0.2	0.6	1.4	0.5
	S032	0.4	0.9	0.4	0.5

*Values are percentages. Species assignments apply to the consensus genotype for the whole cluster. CCG. cluster-consensus genotype. †CCGs with distribution >15%.

### Associations between Species and Reported Manifestations or Produce Consumption

Of the 2,761 evaluable genotyped specimens, we excluded 1,045 (37.8%) that lacked an associated CNHGQ record. Seventeen patients had repeat collections, providing 2 *Cyclospora* specimens each; of those, we excluded 5 patients who exhibited discordant genotypes (10 samples [0.4%]). For the remaining 12 patients, we retained the sample with more complete genotype data (12 exclusions [0.4%]). We also excluded sequences with ambiguous 360i2 data (n = 156 [5.7%]). We used a final sample set of 1,538 patient sequences (55.7% of the evaluable genotyping set) for analyses (n = 214 from 2018, n = 413 from 2019, n = 482 from 2020, n = 429 from 2021). Of the final set, 437 (28.4%) sequences were *C. ashfordi* and 1,101 (71.6%) were *C. cayetanensis*.

Most patients were White (n = 1,048 [94%] of 1,113 with race data), and more were female than male (n = 862 [57%] female, n = 650 male [43%], of 1,502 with sex data). The average age was 50.7 years. We detected no statistically significant difference between species groups (p = 0.10). More patients infected with *C. ashfordi* self-identified as Hispanic (n = 78 [20%]) than those infected with *C. cayetanensis* (n = 105 [12%]; p<0.001) (Table 2). Eight cyclosporiasis clinical manifestations were documented in the CNHGQ (Table 3). Diarrhea was most commonly reported (n = 1,224 [98%]), followed by abdominal cramps (n = 921 [77%]), fatigue (n = 920 [76%]), weight loss (n = 856 [72%]), nausea (n = 829 [69%]), vomiting (n = 823 [68%]), fever (n = 758 [63%]), and anorexia (n = 751 [63%]). More patients infected with *C. cayetanensis* than *C. ashfordi* reported experiencing fatigue (79% vs. 71%; p = 0.003). Among those who responded to all symptom-related questions (n = 1,159), the median number of symptoms in patients with *C. cayetanensis* was 6 and in patients with *C. ashfordi* was 5, although this difference was not statistically significant (p = 0.070). Of the 1,416 patients who responded to questions about hospitalization, 81 (5.7%) were hospitalized a median of 3 nights (interquartile range 2–4 nights); maximum stay was 16 nights. A slightly larger percentage of patients with *C. ashfordi* (n = 30 [7.7%]) were hospitalized than were patients with *C. cayetanensis* (n = 51 [5.0%]), although the difference was not statistically significant (p = 0.053).

**Table 2:** Characteristics of patients in study of *Cyclospora* *genotypic variations and associated epidemiologic characteristics*, United States, 2018–2021*

Characteristic	Overall	Infected with *C. ashfordi*	Infected with *C. cayetanensis*	p value
No. patients	1,538	437	1,101	
Age, y				0.10
Mean (SD)	50.7 (16.6)	49.6 (16.5)	51.1 (16.6)	
Missing, no.	22	8	14	
Race				0.11
White	1,048 (94)	306 (92)	742 (95)	
Black or African American	45 (4.0)	21 (6.3)	24 (3.1)	
Asian	13 (1.2)	3 (0.9)	10 (1.3)	
American Indian or Alaska Native	6 (0.5)	2 (0.6)	4 (0.5)	
Native Hawaiian or Pacific Islander	1 (<0.1)	0 (0)	1 (0.1)	
Missing, no.	425	105	320	
Ethnicity				<0.001
Non-Hispanic	1,080 (86)	310 (80)	770 (88)	
Hispanic	183 (14)	78 (20)	105 (12)	
Missing, no.	275	49	226	
Sex				>0.99
F	862 (57)	244 (57)	618 (57)	
M	640 (43)	181 (43)	459 (43)	
Missing, no.	36	12	24	

*Values are no. (%) except as indicated.

**Table 3:** Patient-reported clinical symptoms and outcomes overall and by *Cyclospora* species exposure *in study of* *Cyclospora* *genotypic variations and associated epidemiologic characteristics*, United States, 2018–2021*

Clinical symptom or outcome	Overall	Infected with *C. ashfordi*	Infected with *C. cayetanensis*	p value
No. patients	1,538	437	1,101	
Diarrhea				0.92
Y	1,224 (98)	365 (98)	859 (98)	
N	26 (2.1)	8 (2.1)	18 (2.1)	
Missing, no.	288	64	224	
Weight loss				0.71
Y	856 (72)	255 (71)	601 (72)	
N	340 (28)	105 (29)	235 (28)	
Missing, no.	342	77	265	
Fever				0.71
Y	758 (63)	231 (64)	527 (63)	
N	445 (37)	131 (36)	314 (37)	
Missing, no.	335	75	260	
Fatigue				0.003
Y	920 (76)	257 (71)	663 (79)	
N	283 (24)	105 (29)	178 (21)	
Missing, no.	335	75	260	
Anorexia				0.86
Y	751 (63)	229 (63)	522 (63)	
N	440 (37)	132 (37)	308 (37)	
Missing, no.	347	76	271	
Nausea				0.076
Y	829 (69)	236 (65)	593 (70)	
N	379 (31)	127 (35)	252 (30)	
Missing, no.	330	74	256	
Vomiting				0.14
Y	823 (68)	258 (71)	565 (67)	
N	380 (32)	103 (29)	277 (33)	
Missing, no.	335	76	259	

*Values are no. (%) except as indicated.

We included 64 produce items for bivariate analysis, although 26.5% of patients (n = 408) did not respond to produce-related questions. A greater percentage of patients infected with *C. ashfordi* recalled eating cilantro (44% vs. 28%; p<0.001), squash (14% vs. 8.9%; p = 0.010), guacamole (31% vs. 23%; p = 0.003), pico de gallo (32% vs. 25%; p = 0.022), plum (8.8% vs. 4.9%; p = 0.015), onion (49% vs. 41%; p = 0.018), mango (18% vs. 13%; p = 0.024), and lemon or lime (43% vs. 35%; p = 0.014) (Table 4). More patients infected with *C. cayetanensis* reported consumption of iceberg lettuce (49% vs. 39%; p = 0.001), cauliflower (23% vs. 11%; p<0.001), bagged salad kit (15% vs. 9.4%; p = 0.013), and carrot (30% vs. 23%; p = 0.018).

**Table 4:** Patient-reported produce consumption in the 2 weeks before symptom onset, overall and by *Cyclospora* species exposure, *in study of* *Cyclospora* *genotypic variations and associated epidemiologic characteristics* United States, 2018–2021*

Produce	Overall	Infected with *C. ashfordi*	Infected with *C. cayetanensis*	p value
No. patients	1,538	437	1,101	
Produce consumed more commonly by patients infected with *C. ashfordi*				
Cilantro				<0.001
Y	356 (33)	145 (44)	211 (28)	
N	725 (67)	183 (56)	542 (72)	
Missing, no.	457	109	348	
Plum				0.015
Y	63 (6.1)	28 (8.8)	35 (4.9)	
N	978 (94)	292 (91)	686 (95)	
Missing, no.	497	117	380	
Lemon or lime				0.014
Y	390 (37)	137 (43)	253 (35)	
N	652 (63)	182 (57)	470 (65)	
Missing, no.	496	118	378	
Mango				0.024
Y	150 (14)	58 (18)	92 (13)	
N	896 (86)	264 (82)	632 (87)	
Missing, no.	492	115	377	
Squash				0.010
Y	110 (11)	46 (14)	64 (8.9)	
N	931 (89)	278 (86)	653 (91)	
Missing, no.	497	113	384	
Onion				0.018
Y	459 (44)	159 (49)	300 (41)	
N	596 (56)	166 (51)	430 (59)	
Missing, no.	483	112	371	
Pico de gallo				0.022
Y	288 (27)	103 (32)	185 (25)	
N	782 (73)	223 (68)	559 (75)	
Missing, no.	468	111	357	
Guacamole				0.003
Y	271 (25)	102 (31)	169 (23)	
N	799 (75)	224 (69)	575 (77)	
Missing, no.	468	111	357	
Produce more commonly consumed among patients infected with *C. cayetanensis*				
Iceberg lettuce				0.001
Y	509 (46)	128 (39)	381 (49)	
N	592 (54)	201 (61)	391 (51)	
Missing, no.	437	108	329	
Bagged salad kit				0.013
Y	142 (13)	30 (9.4)	112 (15)	
N	925 (87)	290 (91)	635 (85)	
Missing, no.	471	117	354	
Carrot				0.018
Y	298 (28)	73 (23)	225 (30)	
N	779 (72)	248 (77)	531 (70)	
Missing, no.	461	116	345	
Cauliflower				<0.001
Y	202 (19)	34 (11)	168 (23)	
N	865 (81)	289 (89)	576 (77)	
Missing, no.	471	114	357	

*Values are no. (%) except as indicated. We included produce items with statistically significant differences by χ^2^ test.

In addition to covariates for time and geography, we included covariates for ethnicity and produce items with statistically significant results (Table 4) in a GEE model with *Cyclospora* species as the outcome. We dropped patients with missing covariate values, leaving 908 patients (59%) for analysis (*C. ashfordi*, n = 277; *C. cayetanensis*, n *=* 631). The species proportions remained similar even with such exclusions, suggesting an equitable distribution of missing data across species. The model included year and ordinal date of illness onset to control for temporal variations in species prevalence, but we did not interpret those data for analysis. We detected no multicollinearity among the predictor variables.

**Figure 4:**
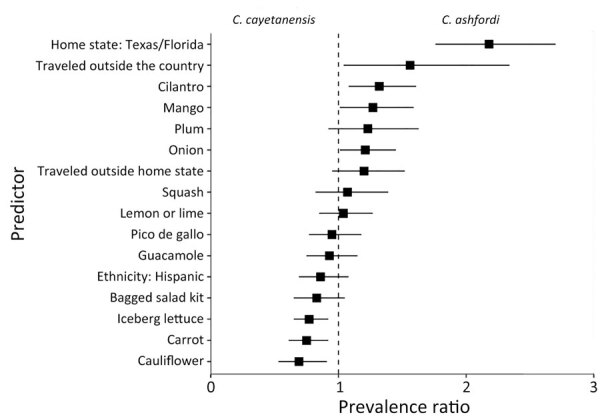
Forest plot presenting prevalence ratio point estimates for all predictors in the generalized estimating equation model *in study of* *Cyclospora* *genotypic variations and associated epidemiologic characteristics* United States, 2018–2021. Prevalence ratios are determined with *C. ashfordi* as the numerator and *C. cayetanensis* as the denominator, illustrating the comparative prevalence of these species across the various predictors. Boxes represent prevalence ratio point estimates; whiskers, 95% CI. The dashed line represents a prevalence ratio of 1.

Geography and the produce items cilantro, mango, onion, iceberg lettuce, carrot, and cauliflower all had a statistically significant association with *Cyclospora* species, all else being equal (Figure 4). In patients from Texas or Florida, prevalence of infection with *C. ashfordi* was 2.18 (95% CI 1.76–2.70) times greater than for *C. cayetanensis*. Patients reporting international travel were also more frequently infected with *C. ashfordi* (prevalence ratio [PR] 1.56 [95% CI 1.04–2.34]) than were those who remained in-country. Infections with *C. ashfordi* were associated with consumption of cilantro (PR 1.32 [95% CI 1.08–1.61]), mango (PR 1.27 [95% CI 1.01–1.59]), and onion (PR 1.45 [95% CI 1.01–1.21]), whereas consumption of iceberg lettuce (PR 0.77 [95% CI 0.65–0.92]), carrot (PR 0.75 [95% CI 0.61–0.92]), and cauliflower (PR 0.69 [95% CI 0.53–0.91]) were significantly associated with *C. cayetanensis* infections.

## Discussion

This study used a novel genotype designation to understand the genetic diversity and molecular epidemiologic trends of cyclosporiasis in the United States over 4 consecutive years. The data we used are likely representative of the US cyclosporiasis trends given our relatively large and diverse sample size, including samples collected over multiple years.

Although there are annual cyclosporiasis outbreaks in the United States, our understanding of the transmission dynamics of *Cyclospora* is limited. One such knowledge gap is where produce originally becomes contaminated. One hypothesis suggests that contamination arises from local prevalence or persistence of *Cyclospora* within the United States (*17*). Other theories posit that sources of contamination originate outside the United States from repeated introductions such as imported produce, migrant or seasonal farmworkers, travelers carrying the parasite, or other yet-to-be-investigated means (*17*,*18*).

This study detected high heterogeneity of CCGs, both over time and by state, findings that may support the hypothesis that cases of cyclosporiasis in the United States are likely caused by frequent new introductions, rather than *Cyclospora* parasites that persist in the local environment. For example, the high heterogeneity of CCGs observed by state and over time could be attributable to imported produce. Given the globalization of food supplies, produce sold in the United States are imported from many areas, some of which may be endemic for *Cyclospora*, at different times, which might explain the diversity of CCGs we reported.

We found that New York, Texas, and Florida tended to have higher numbers of reported cyclosporiasis cases than other states. The reasons for this observation are outside the scope of this study but could be attributed to population size or myriad differences related to public health reporting requirements and investigation capacities, access to healthcare, availability of diagnostic testing, or healthcare provider knowledge. For those same reasons, there is an inherent potential for sampling bias to occur in surveillance data. Although those 3 states saw less-pronounced shifts in *Cyclospora* genetic diversity, we observed no clear dominant or persistent pattern either at the species or CCG level, contrary to what we would expect if the parasite were persisting in the local environment.

The separation of *C. cayetanensis* into 3 species is a recent taxonomic change (*7*) and is yet to gain widespread acceptance, as is typical for recent taxonomic revisions, particularly for medically important pathogens. We chose to retain the use of those proposed species’ names for the purposes of this article. Another main finding of this analysis is that the 2 *Cyclospora* species described previously (*7*) showed distinct epidemiologic characteristics, potentially underpinning some presently unknown biologic differences between *C. cayetanensis* and *C. ashfordi* and providing further evidence in support of their taxonomic separation. Certain produce items were more commonly associated with *C. cayetanensis* and others with *C. ashfordi.* Because numerous specimens were collected during outbreak investigations, it is likely that related specimens will be found within clusters that are determined through epidemiologic analysis, with one food source serving as a common link. The occurrence of such clustering can create spurious associations; we used a multivariate GEE model to establish the relationship between predictor variables and *Cyclospora* species while considering the correlation between specimens within the same cluster (*19*). To account for spatiotemporal variability in CCG distribution, the model included covariates for geography and year. Our findings demonstrated that associations between some produce items and species remained statistically significant even after adjusting for clustering and spatiotemporal variance. However, certain produce items may be underreported because they do not constitute a major ingredient in a dish and persons may not recall consuming them; herbs are a prime example. In addition, because many fresh produce items are consumed in mixtures such as bagged salad mixes, guacamole, or pico de gallo, it can be difficult to pinpoint the true vehicles or predictors for *Cyclospora*. Despite those drawbacks, our findings identified several produce items of interest, and determining if there are factors in growing, harvesting, handling, or storage conditions of those produce items that may increase the likelihood of *Cyclospora* contamination is an area for further research.

Given that CCGs are an emergent property of probabilistic genetic clustering and the genetic clusters themselves are composed of similar but not always identical genotypes (*5*,*6*), the CCGs reported here may change in future studies. For example, a set of isolates may always genetically cluster together, but their CCG may change if isolates added or removed from the dataset cause the consensus genotype of the genetic cluster to change. Furthermore, the CCGs we described were created using 8 genetic markers; future addition of genotyping markers will increase the discriminatory capacity and long-term stability of CCGs but will also lead to the redefinition of genetic clusters. For those reasons, the CCGs we identified are robust for this study but may evolve when using either additional markers or other datasets, and the produce relationships identified in these analyses may change in future analyses. Overall, this study provides a foundation for improving our understanding of cyclosporiasis epidemiology in the United States. 

The CNHGQ surveys were administered to patients after a confirmed laboratory diagnosis of cyclosporiasis, typically many days after infection has occurred. Therefore, the data may be affected by recall or symptom-associated biases (*20*), missing data, discrepancies, or errors. For this study, more than one third of samples from the genotyping dataset lacked corresponding CNHGQ data. Given their contributions to this type of molecular epidemiology study, having more complete CNHGQ information should be a focus for future work.

This study highlights several opportunities for future work. First, the CCGs were determined using at most 8 genetic markers that may not capture differences in other regions of the *Cyclospora* genome. Therefore, including additional genotyping markers could enhance the use of CCGs for molecular epidemiologic studies. Second, there is a need to collect and sequence *Cyclospora* samples from endemic areas because those data will enhance the understanding of the genetic diversity of *Cyclospora* and CCGs over space and time, and their genetic information may improve the stability of CCGs and genotyping methods.

Understanding food distribution networks and their variations can offer valuable insights into why specific species and CCGs appear in certain locations during particular times of year; that knowledge could shed light on whether CCGs are associated with the origin, processing or distribution of produce. Continued genotyping as part of ongoing, routine cyclosporiasis surveillance will bolster knowledge on temporal patterns of *Cyclospora*. Overall, such studies promise to improve cyclosporiasis outbreak investigations, potentially enabling investigators to trace the origins of *Cyclospora*-contaminated produce with heightened precision and fostering the development of prevention and control programs.

Appendix Additional information about *Cyclospora* genotypic variations and associated epidemiologic characteristics, United States, 2018–2021.
