# Supplementary material for: Cyclospora Genotypic Variations and Associated Epidemiologic Characteristics, United States, 2018–2021
Source: Emerg Infect Dis. 2025 Feb;31(2):256–66. doi: 10.3201/eid3102.240399 (PMC11845137; doi:10.3201/eid3102.240399)
Supplement: Appendix — Additional information about Cyclospora genotypic variations and associated epidemiologic characteristics, United States, 2018–2021. [file 24-0399-Techapp-s1.pdf]

Article DOI: https://doi.org/10.3201/eid3102.240399

EID cannot ensure accessibility for supplementary materials supplied by authors. Readers who have difficulty accessing supplementary content should contact the authors for assistance.

Cyclospora Genotypic Variations and Associated Epidemiologic Characteristics, United States, 2018–2021

Appendix

Appendix Table 1. Proportion of *Cyclospora* specimens received from states that were either *C. cayetanensis* or *C. ashfordi*, United States, 2018–2021*

State	Year	Infected with *C. cayetanensis*, no. (%)	Infected with *C. ashfordi*, no. (%)
Florida (n = 222)	2019	11 (84.6)	2 (15.4)
	2020	19 (21.1)	71 (78.9)
	2021	65 (54.6)	54 (45.4)
Georgia (n = 107)	2018	4 (100.0)	0 (0.0)
	2019	23 (63.9)	13 (36.1)
	2020	23 (65.7)	12 (34.3)
	2021	26 (81.3)	6 (18.8)
Illinois (n = 212)	2018	101 (91.0)	10 (9.0)
	2019	6 (50.0)	6 (50.0)
	2020	54 (74.0)	19 (26.0)
	2021	13 (81.3)	3 (18.8)
Iowa (n = 185)	2018	61 (85.9)	10 (14.1)
	2019	22 (81.5)	5 (18.5)
	2020	84 (96.6)	3 (3.4)
Massachusetts (n = 85)	2019	33 (38.8)	52 (61.2)
Michigan (n = 54)	2019	6 (19.4)	25 (80.6)
	2021	17 (73.9)	6 (26.1)
Minnesota (n = 204)	2018	32 (88.9)	4 (11.1)
	2019	18 (60.0)	12 (40.0)
	2020	86 (90.5)	9 (9.5)
	2021	38 (88.4)	5 (11.6)
Nebraska (n = 76)	2020	50 (87.7)	7 (12.3)
	2021	17 (89.5)	2 (10.5)
New York (n = 651)	2018	44 (65.7)	23 (34.3)
	2019	210 (70.7)	87 (29.3)
	2020	75 (68.2)	35 (31.8)
	2021	135 (76.3)	42 (23.7)
Tennessee (n = 31)	2020	13 (100.0)	0 (0.0)
	2021	16 (88.9)	2 (11.1)
Texas (n = 551)	2018	59 (60.2)	39 (39.8)
	2019	88 (40.2)	131 (59.8)
	2020	48 (28.6)	120 (71.4)
	2021	48 (72.7)	18 (27.3)
Wisconsin (n = 224)	2018	120 (97.6)	3 (2.4)
	2019	13 (43.3)	17 (56.7)
	2020	25 (80.6)	6 (19.4)
	2021	36 (90.0)	4 (10.0)

*Data for several states, including AZ, CA, CT, IN, KS, LA, MD, MO, MT, NH, NJ, NM, ND, OH, OK, PA, RI, SC, SD, UT, and VA are not shown because the number of specimens submitted during the study period was <20.

Appendix Table 2. Distribution of cluster-consensus genotypes (CCG) in the analytic sample.

CCG #	Proportion of analytic sample, no. (%)
S001	243 (8.8)
S002	49 (1.8)
S003	59 (2.1)
S004	408 (14.8)
S005	58 (2.1)
S006	170 (6.2)
S007	256 (9.3)
S008	89 (3.2)
S009	40 (1.4)
S010	21 (0.8)
S011	47 (1.7)
S012	332 (12)
S013	19 (0.7)
S014	19 (0.7)
S015	25 (0.9)
S016	52 (1.9)
S017	25 (0.9)
S018	26 (0.9)
S019	88 (3.2)
S020	98 (3.5)
S021	50 (1.8)
S022	125 (4.5)
S023	79 (2.9)
S024	31 (1.1)
S025	29 (1.1)
S026	9 (0.3)
S027	28 (1)
S028	33 (1.2)
S029	167 (6)
S030	16 (0.6)
S031	20 (0.7)
S032	15 (0.5)
S033	35 (1.3)

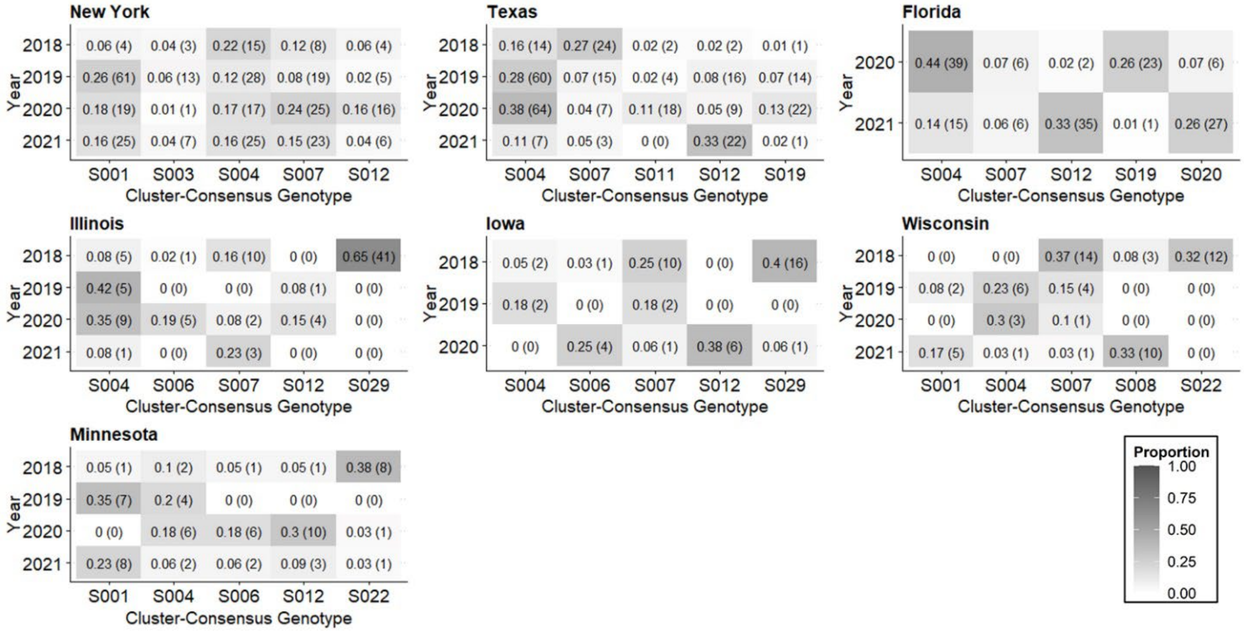

Appendix Figure. Heatmaps illustrating the proportions of the top 5 most prevalent CCGs in each of the 7 states included in this analysis throughout the study period, excluding specimens related to outbreak clusters. The proportions are calculated as a percentage of the total number of specimens within a state

for a given year. Darker shades represent higher proportions. The total number of specimens submitted per year is as follows: New York—67 (2018), 236 (2019), 103 (2020), 157 (2021); Texas—88, 213, 168, 66; Florida—0, 0, 89, 105; Illinois—63, 12, 26, 13; Iowa—40, 11, 16, 0; Wisconsin—38, 26, 10, 30; Minnesota—21, 20, 33, 35.
